# CD146 promotes resistance of NSCLC brain metastases to pemetrexed via the NF-κB signaling pathway

**DOI:** 10.3389/fphar.2024.1502165

**Published:** 2025-01-13

**Authors:** Hao Qu, Yan Fang, Feng Zhang, Wenwen Liu, Shengkai Xia, Wenzhe Duan, Kun Zou

**Affiliations:** ^1^ Department of Radiation Oncology, The First Affiliated Hospital, Dalian Medical University, Dalian, Liaoning, China; ^2^ Department of Radiation Oncology, The Second Affiliated Hospital, Dalian Medical University, Dalian, Liaoning, China; ^3^ Department of Respiratory Medicine, The Second Affiliated Hospital, Dalian Medical University, Dalian, Liaoning, China

**Keywords:** CD146, brain metastasis, chemotherapeutic resistance, pemetrexed, lung cancer

## Abstract

**Introduction:**

Pemetrexed is a first line drug for brain metastases from lung cancer, either as monotherapy or combined with other drugs. The frequent occurrence of initial and acquired resistance to pemetrexed results in limited treatment effectiveness in brain metastases. CD146 was recently found to play important roles in chemoresistance and tumor progression. However, the underlying mechanisms of CD146’s effects in pemetrexed resistance remain undefined.

**Method and results:**

Sensitivity to pemetrexed was assessed with a preclinical brain metastasis (BM) model based on lung adenocarcinoma PC9 cells. The role and mechanism of CD146 in pemetrexed resistance in non-small cell lung cancer (NSCLC) brain metastasis were explored *in vitro* and *in vivo*. A subpopulation of brain metastatic cells derived from progenitor PC9 cells (PC9-BrMS) was significantly resistant to pemetrexed. CD146 levels were significantly increased in pemetrexed resistant brain metastases, while CD146 inhibition suppressed pemetrexed resistance in BM cells. Mechanistically, CD146 mediated pemetrexed resistance in brain metastatic cells by promoting DNA damage repair, maintaining normal cell cycle progression, and regulating the NF-KB pathway to counter apoptosis, and these effects was based on increased DNA damage, cell cycle arrest, and occurrence of apoptosis after CD146 inhibition as well as the reemergence of pemetrexed resistance after CD146 restoration.

**Discussion:**

In summary, this study revealed that the resistance of NSCLC brain metastatic cells to PEM was dependent on CD146.Thus CD146 might be targeted in clinic to overcome pemetrexed resistance in brain metastases from NSCLC.

## Introduction

Non-small cell lung cancer (NSCLC), the prevalentpathological type of lung cancer, easily develops distant metastases, with Brain metastases (BM) account for 40% of all metastases and constitute the main cause of death in NSCLC cases (Steeg et al., 2011; Sorensen et al., 1988). Individuals with BM have very low survival, with median survival of only 4–6 months (Cheng and Perez-Soler, 2018; Boire et al., 2020; Zhu et al., 2022). Although the treatment outcome is not significant, currently feasible treatment modalities combine surgery, radiation therapy, chemotherapy, targeted therapy, and/or antiangiogenic therapy (Shi et al., 2017). However, these approaches often face limitations, including the blood-brain barrier (BBB) that restricts the delivery of therapeutic agents to the central nervous system. Additionally, the heterogeneity of metastatic tumors and their variable response to treatment further complicate management strategies. Although progress has been made in understanding the biology of brain metastasis, with the discovery of many predictive biomarkers (Zou et al., 2023; Xie et al., 2024) and new mechanisms related to drug resistance (Fu et al., 2024) through innovative techniques such as single-cell sequencing, effective treatment is still difficult to achieve. This highlights the urgent need for innovative treatment strategies and a deeper understanding of the potential mechanisms that lead to such devastating complications.

Since indications for surgery and radiation therapy are very limited, drug therapy based mainly on chemotherapy is required in individuals with BM (Ettinger et al., 2019; Yousefi et al., 2017). Pemetrexed (in combination with cisplatin or carboplatin) represents a firstline drug for the treatment of lung adenocarcinoma per the National Comprehensive Cancer Network for NSCLC guidelines (version 2.2022) (Horbinski et al., 2023). However, treatment outcome in the BM population is not very good (Kim, 2016; Yang et al., 2020). In-depth studies of chemotherapy resistance mechanisms may provide insights into the design of new therapeutic approaches for BM cases. Tumor resistance to pemetrexed arises from various factors, such as the upregulation of folatedependent enzymes, such as thymidine synthase (TS) (Buque et al., 2012; Gonen and Assaraf, 2012). Furthermore, alterations in the expression levels of key enzymes in the *de novo* synthesis pathway of thymidine and purine nucleotides also impact the sensitivity of tumors to pemetrexed (Yousefi et al., 2017; Horbinski et al., 2023). Thus, pemetrexed can inhibit tumor growth by preventing DNA replication and inducing cell cycle arrest (Backus et al., 2000). Additionally, pemetrexed inhibits tumors by promoting DNA damage (Ding et al., 2020) and inducing apoptosis (Chen et al., 2020; Lehmann et al., 1987). Chemotherapy resistance in tumors is usually achieved through these pathways. However, the upstream regulatory mechanisms of DNA damage repair and apoptosis reduction in BMassociated drug resistance need to be further examined.

CD146 represents a cell adhesion molecule firstly described as melanomaspecific that belongs to the immunoglobulin superfamily (Wang and Yan, 2013). CD146 also regulates a variety of cellular events such as cell invasion (Liang et al., 2022), migration (Liu et al., 2021), angiogenesis (Zhang et al., 2022), epithelialmesenchymal transition (EMT) (Liu et al., 2022), immune responses (Duan et al., 2021) and signal transduction (Wang et al., 2020). It has been shown that differential expression of CD146 in primary tumors is associated with metastatic potential and low survival in a variety of tumors, demonstrating its great potential in cancer therapy (Oka et al., 2012; Zeng et al., 2012). Although CD146 is known to mediate cisplatin resistance in NSCLC (Tripathi et al., 2017), its expression and roles in brain metastasis from NSCLC are undefined. Many previous reports have reported that CD146 regulates the NFκB pathway in different cancers (Zeng et al., 2014; Ruma et al., 2016; Zheng et al., 2016), but this has not been confirmed in NSCLC brain metastases. To investigate the mechanism of the resistance of lung cancer brain metastases to pemetrexed, we further investigated the effects of CD146 and its overexpression on chemotherapy resistance and cellular functions in NSCLC brain metastases by gene knockout assay.

## Materials and methods

### Cell culture

Human lung cancer PC9 cells were provided by the Chinese Academy of Medical Sciences (China). Brain metastatic subpopulations (PC9BrM1, PC9BrM2, and PC9BrM3) were built by infusing PC9 cells in immunodeficient mice through left ventricular injection and separating metastatic cells from collected brain metastases (Liu et al., 2019). Highly brain metastatic lung cancer PC9BrM3 cells were obtained through repeated injection separation expansion cycling three additional times, and maintained in RPMI 1640 containing 10% fetal bovine serum (FBS), 100 U/mL penicillin and 100 U/mL streptomycin at 37°C in a humid environment with 5% CO_2_.

I verified that all human cell lines have been authenticated through STR (or SNP) profiling within the past 3 years, and all experiments were conducted using mycoplasma-free cells.

### Antibodies

Antibodies against CD146 (661531lg) GAPDH (60041lg), BCL2 (601781lg) and BAX (505992lg) were provided by Proteintech (China). Antibodies targeting γH2AX (#9718), cleaved caspase 3 (#9664), cleaved PARP (#5625), phosphoNFκBp65 (#3033), NFκB (#8242), phosphoIkBα(#2859)and IkBα(#4814)were from Cell Signaling Technology.

### Cell viability and colony formation assay

Cell viability was monitored with CCK8 kit (CCK8; K1018, ApexBio, United States) following the manufacturer suggestions. In a 96well plate, cells were seeded at 5,000/well for overnight incubation. Then, the treatments were removed and replaced by specific drugs for 72 h. After 72 h of culturing, the culture medium was enriched with 10% CCK8 and subsequently incubated for an additional 2 h. The obtained data (optical density) were assessed using a Multiskan™ FC Microplate Reader from Thermo Fisher Scientific at a wavelength of 450 nm.

For the colony formation assay, cells underwent seeding into a 6well plate at 1,000/well, followed by incubation at 37°C and 5% CO_2_ with the medium refreshed every other day. Following a 2 weeks incubation period, 4% paraformaldehyde was used for fixation, with subsequent staining with 4% crystal violet for 30 min.

### Transwell migration assay

The migration assay used Transwell chambers (Corning, New York, United Kingdom). Tumor cells (1 × 10^4^/well) were plated into chambers and coincubated with 200 µL of serumfree medium with or without pemetrexed. Totally 72 h later, fixation was carried out with 4% paraformaldehyde with subsequent crystal violet staining. Photomicrographs were obtained. Experimental data were statistically assessed with ImageJ.

### Immunoblot

Total protein extraction from harvested cells was carried out with RIPA cell lysis buffer that contained protease (Meilunbio, China) and phosphatase (Sigma, United Kingdom) inhibitor cocktails. Protein concentration was assessed with the BCA Protein Assay Kit (Thermo Fisher). Proteins were then separated by SDSPAGE and electrotransferred onto a nitrocellulose membrane with a miniProtean electrophoresis system (Millipore, United States). After a 2 h blocking with Trisbuffered saline supplemented with 0.1% Tween20 (TBST) and 5% skimmed milk, the membranes underwent successive incubations with primary and secondary antibodies. The ECL kit (Tanon, China) was utilized for detection. ImageJ was utilized for quantifying protein bands (National Institutes of Health, United Kingdom). All experiments are conducted by at least three independent researchers.

### Silencing and overexpression of CD146

CD146targeting shRNA and the respective negative control shRNA (shNC) were built in a lentiviral vector provided by GeneChem (China). The target sequence of the CD146 shRNA was 5′GAG​CGA​ACT​TGT​AGT​TGA​A3′. 293T cells were infected with shRNAexpressing lentivirus utilizing Lipofectamine 2000 (Invitrogen, United States) per the manufacturer’s protocol.

Infection with the shRNAexpressing lentivirus and establishment of stably infected single clones were carried out as directed by the manufacturer. Immunoblot was carried out to assess knockdown efficiency. Transfectionpositive cells were screened with complete medium containing puromycin (1 μg/mL) and cultured continuously to ensure stable gene expression in cells. CD146 overexpression lentivirus (OECD146) was purchased from the GeneChem Company.

### Flow cytometry

For apoptosis assay, cells after transfection with CD146 shRNA and control shRNA (10^6^ cells) underwent culture in 6well plates and treatment with PEM (80 nM) for 72 h prior to cell collection. According to the manufacturer’s instructions, cell staining was carried out with AnnexinVAPC and 7aminoactinomycin D (7AAD) (ECKA218, Elabscience). After staining, flow cytometry (BD Accuri TM C6 cytometer) was employed for analysis using Flowjo.

For cell cycle assays, flow cytometry was carried out with a cell cycle kit (no. C1052; Beyotime, China) per the manufacturer’s protocol, and data analysis utilized the ModFit software.

### Immunofluorescence staining

Tumor cells underwent seeding in a 48 well plate at 10,000 per well for quantitating γH2AX. Pemetrexed was administered for 48 h, followed by fixation with 4% paraformaldehyde (KeyGEN BioTECH) for 20 min with addition of 0.25% Triton X100 for permeabilization for 810 min. Blocking was performed with 3% BSA (Sigma) at ambient for 60 min. Primary antibody targeting γH2AX (1:400) was added for overnight incubation at 4°C. Then, cells were stained with Alexa Fluor 594 (1:100; Proteintech) for 1 h followed by DAPI (1:1,000, KeyGEN Biotech) counterstaining for 10 min at ambient shielded from light. A fluorescence microscope (Nikon TE200, Japan) was utilized for image acquisition, and ImageJ was utilized for quantitation.

### Mouse xenograft models

Athymic female BALB-c-nu mice, aged 4–6 weeks, were purchased from Beijing Vital River Laboratory Animal Technology Co., Ltd. and were kept in pathogen-free conditions. The highly brain metastatic subpopulation PC9BrM3 with negative control shRNA (shNC) or CD146 silencing (shCD146) (5 × 10^6^ in 100 µL PBS) was administered by subcutaneous (s.c.) injection to the dorsal left flank of 4 weekold BALB/c nude mice (n = 10/group). Each group was further subdivided into the control and dosing groups (n = 5). Interventional treatment was performed after the tumor reaches 100,150 mm^3^. Control mice were administered DMSO by intraperitoneal injection, and the dosing group was administered 100 mg/kg pemetrexed by intraperitoneal injection once weekly. Tumor volumes were measured at 3 day intervals. The mice were euthanized on the 24th day of treatment, and the tumors were excised for further experiments. The euthanasia was performed via intraperitoneal injection of sodium pentobarbital (200 mg/kg), with death confirmed by respiratory and cardiac arrest, as well as pupil dilation. Humane endpoints such as tumor ulceration, tissue swelling, and cachexia were monitored, and none of the mice in the study exhibited these conditions.

All animals were handled per the recommendations of the Dalian Medical University Animal Research and Care Committee, and studies were performed based on the Dalian Medical University Animal Experimentation Guidelines.

### Immunohistochemistry

Immunohistochemistry (IHC) was carried out to detect γH2AX, BAX, cleaved caspase 3 in mouse subcutaneous tumor tissues. Paraffinembedded tumor tissue sections underwent deparaffinization, hydration, and antigen retrieval. After treatment with 3% hydrogen peroxidemethanol for 20 min, blocking was performed with 5% goat serum albumin for 30 min. Then, incubation was carried out with antibodies targeting mouse BAX (1:100), cleaved caspase 3 (1:100) and γH2AX (1:400) overnight at 4°C. Next, the corresponding secondary antibody was added for a 20 min incubation at ambient. Then, staining was performed with the diaminobenzidine kit. Positive cells were detected microscopically and ImageJ was utilized for analysis.

### Statistical analysis

Quantitative data are mean ± standard deviation (SD) from at least 3 replicates. GraphPad Prism 8.0.2 (GraphPad Software, United States) was used for data analysis. Group pairs and multiple groups were compared by the t-test and one way ANOVA (analysis of variance) with *post hoc* Tukey’s test. An effect was considered statistically significant with a *p*-value below 0.05.

## Results

### CD146 is upregulated in NSCLC brain metastatic cell lines resistant to pemetrexed

**FIGURE 1:**
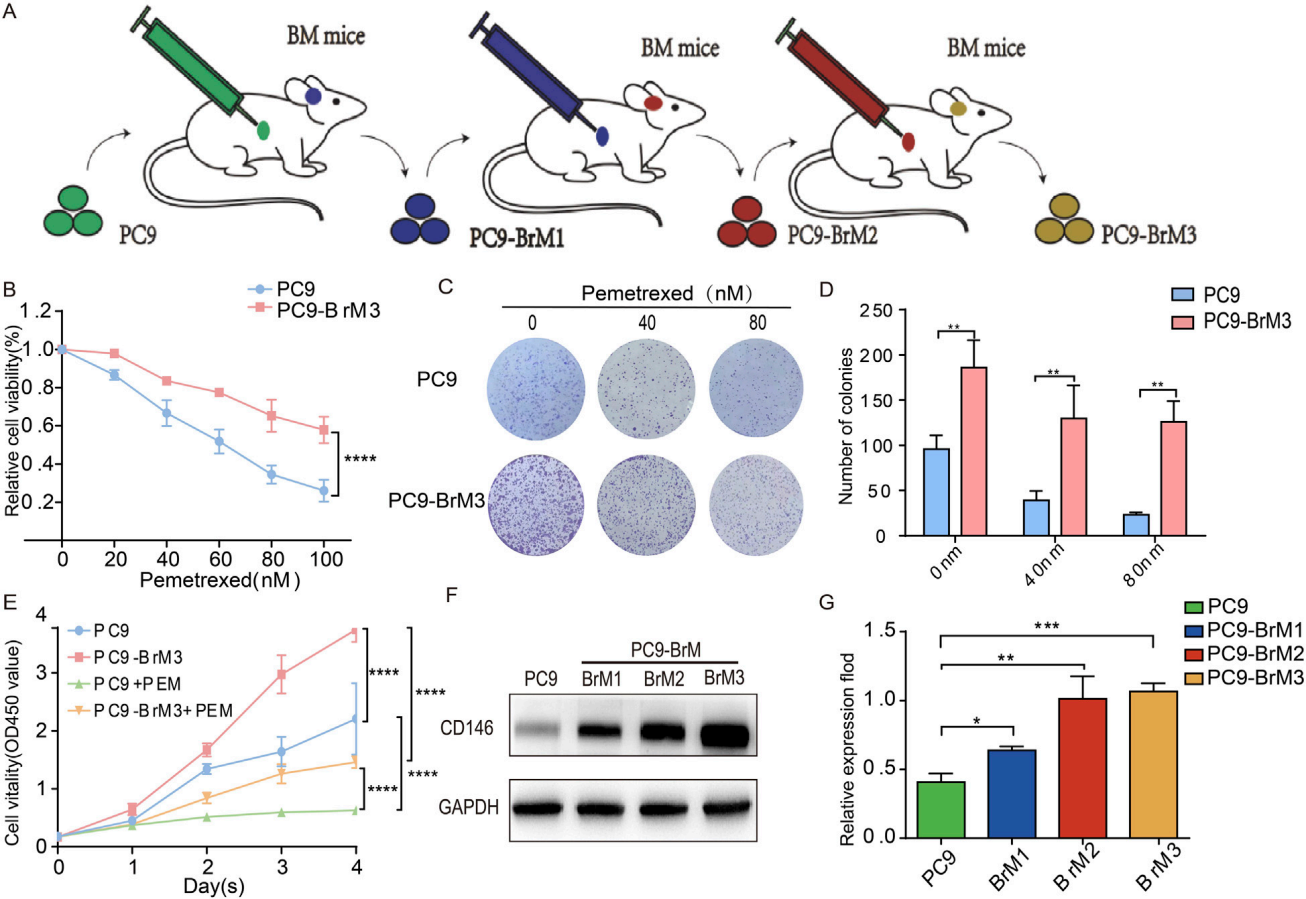
CD146 is upregulated in NSCLC brain metastatic cell lines resistant to pemetrexed. **(A)** Schematic representation of metastatic cells (BrM1, BrM2 and BrM3) isolated from PC9 lung adenocarcinoma cell lines *in vivo*. **(B)** PC9 and PC9BrM3 cells were administered increasing PEM concentrations as indicated for 72 h, and cell viability was assessed with the CCK8 assay. **(C, D)** Colonies from PC9 and PC9BrM3 cells administered PEM (0, 40, or 80 nM) were counted. **(E)** Cell viability after PEM (80 nM) treatment in PC9 and PC9BrM3 cells for various durations (Day 1 Day 4). **(F, G)** Western blot showing CD146 protein amounts in PC9, BrM1, BrM2, and BrM3 cells (*P < 0.05, **P < 0.01, ***P < 0.001).

We used the human lung cancer cell line PC9 and injected it intracardially into immunodeficient mice to isolatebrain metastatic subpopulations. From these, we isolated PC9 clones with higher metastatic potential. After two rounds of *in vivo* screening and *in vitro* expansion, we obtained a series of brain metastatic subpopulations derived from the parental PC9 cells (Figure 1A). We previously showed that PC9BrM3 cells have high propensity to metastasize to the brain (Liu et al., 2019). Additionally, these cells have consistent genetic homology, which rules out heterogeneity between individual cases, and are an ideal model for drug sensitivity/resistances studies.

To compare parental PC9 cells and highly brain metastatic PC9BrM3 cells, these cells were administered pemetrexed. Cell viability and colony formation assays revealed obvious pemetrexed drug resistance of the highly brain metastatic PC9BrM3 compared with the parental PC9 group (Figures 1B–E).

CD146 is a multifunctional molecule produced by multiple cancers. To assess CD146 expression in brain metastatic NSCLC cells, its protein amounts were quantitated. Western blot showed that CD146 was markedly upregulated in brain metastatic cells in comparison with parent cells (Figures 1F, G).

### CD146 enhances pemetrexed tolerance in NSCLC brain metastases *in vitro*

**FIGURE 2:**
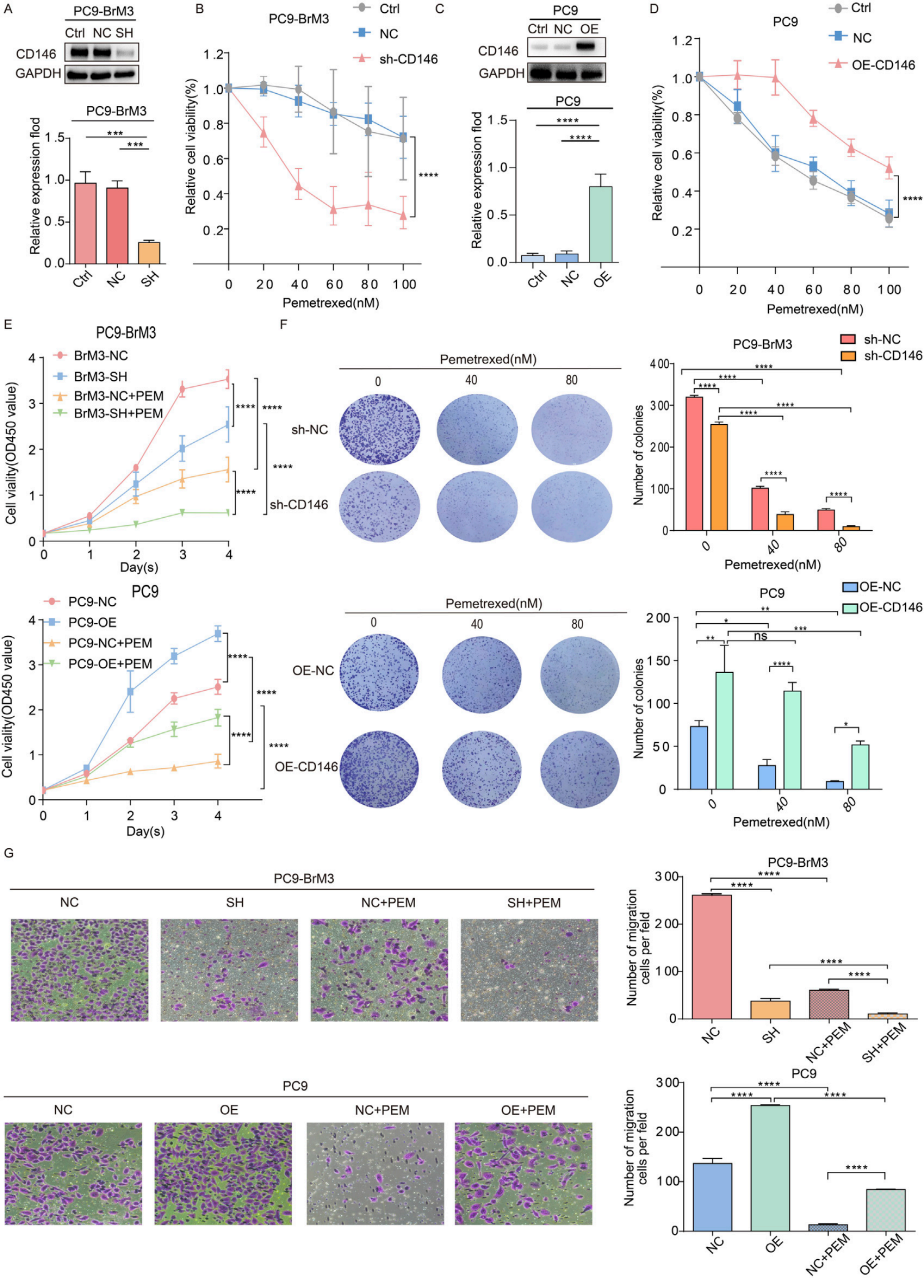
CD146 enhances pemetrexed tolerance in NSCLC brain metastases *in vitro*. **(A, C)** Immunoblot showed shRNA effectively silenced CD146 in PC9BrM3 cells as well as CD146 overexpression in PC9 cells. **(B, D)** CD146 knockdown PC9BrM3 or CD146 overexpressing PC9 cells were administered increasing PEM concentrations as indicated for 72 h, followed by the CCK8 assay. **(E)** Cell viability after treatment with PEM in CD146 knockdown PC9BrM3 or CD146 overexpressing PC9 cells for various times (80 nM, Day 1 Day 4). **(F)** Numbers of colonies formed by CD146 knockdown PC9BrM3 or CD146 overexpressing PC9 cells administered certain concentrations of PEM (0, 40, or 80 nM). Quantitative pixel density analysis of clone formation experiments is shown as a histogram. **(G)** The migration assay was carried out after PEM (80 nM) treatment in CD146 knockdown PC9BrM3 or CD146overexpressing PC9 cells for 72 h. Quantitative pixel density analysis of the migration assay is shown as a histogram (*P < 0.05, **P < 0.01, ***P < 0.001).

Because CD146 is highly expressed in BM, we evaluated the potential link between upregulated protein expression and acquired pemetrexed resistance in brain metastatic cells. shRNA was employed to knockdown CD146, and PC9BrM3 cell proliferation was examined after pemetrexed administration. Cell viability assay showed that inhibition of CD146 with shRNA in BM cells increased PEM sensitivity (Figures 2A, B), whereas CD146 overexpression promoted PC9 cell resistance to PEM (Figures 2C, D). In addition, the proliferative ability of PC9BrM3 cells was altered after CD146 knockdown or overexpression, including after pemetrexed administration (Figure 2E). Conversely, in the colony formation assay, CD146 knockdown significantly decreased PEM resistance in BM cells. Correspondingly, CD146 overexpression in PC9 cells increased PEM resistance (Figure 2F). In addition, the migration assay demonstrated silencing or overexpression of CD146 affected the migratory ability of PC9BrM3 cells, and pemetrexed sensitivity was also significantly affected (Figure 2G). Taken together, these results suggested CD146 expression is positively associated with resistance to PEM in BM cells.

### CD146 maintains cell cycle progression and enhances DNA repair

**FIGURE 3:**
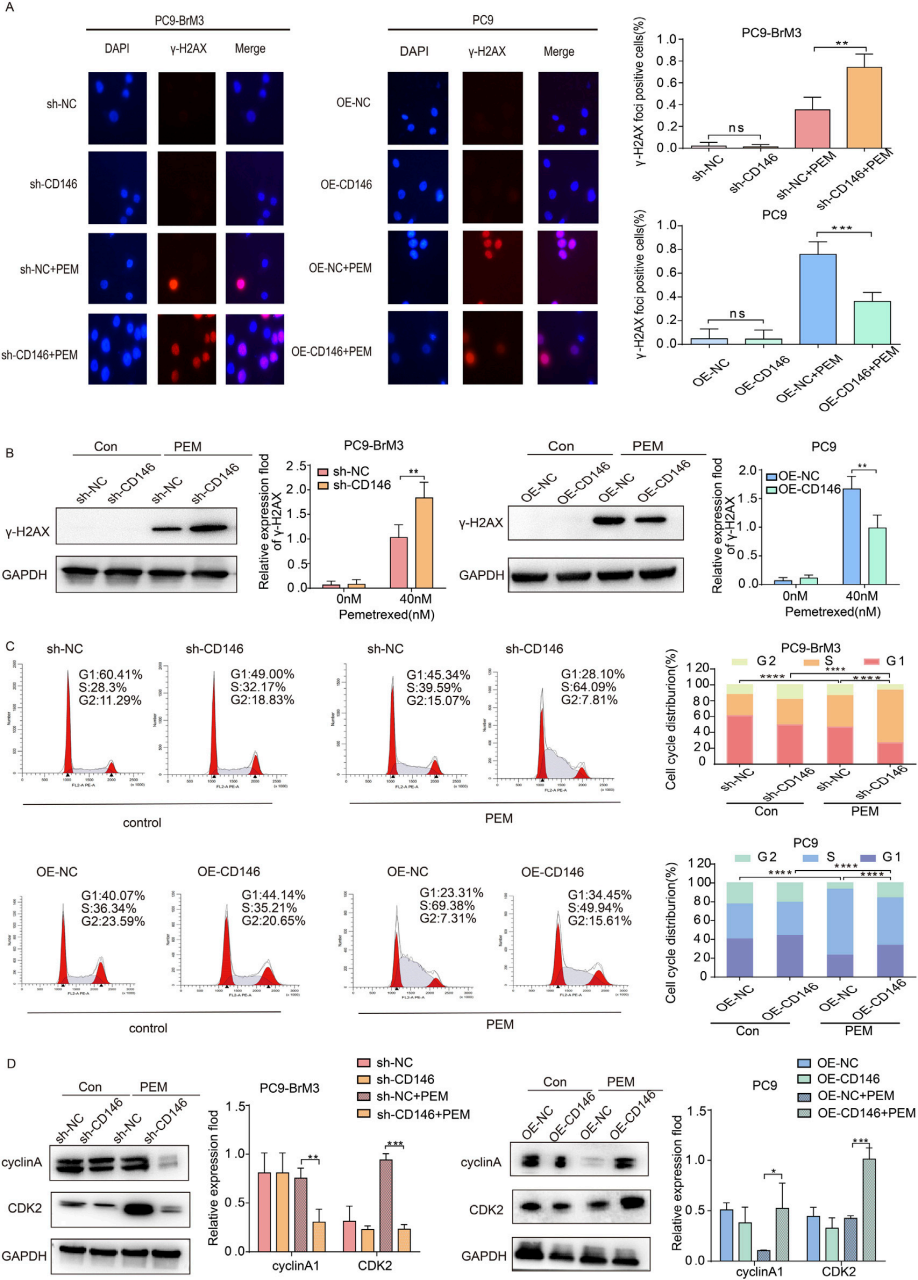
CD146 maintains cell cycle progression and enhances DNA repair. **(A)** Representative immunofluorescence micrographs showing γH2AX (red) and DAPI (blue) in CD146 knockdown PC9BrM3 or CD146overexpressing PC9 cells administered PEM (80 nM) for 72 h. Scale bar, 50 μm. Positivity was indicated by > 5 foci per nucleus. **(B)** Immunoblot showing γH2AX protein expression in CD146 knockdown PC9BrM3 or CD146 overexpressing PC9 cells treated with or without PEM (80 nM) for 72 h. **(C)** Flow cytometry depicting cell cycle changes in CD146 knockdown PC9BrM3 or CD146 overexpressing PC9 cells treated with or without PEM (80 nM) for 72 h. **(D)** Immunoblot showing cyclinA1 and CDK2 protein amounts in CD146 knockdown PC9BrM3 or CD146 overexpressing PC9 cells treated with or without PEM (80 nM) for 72 h (*P < 0.05, **P < 0.01, ***P < 0.001).

Previous studies have shown that pemetrexed can mediate DNA damage in NSCLC (Ding et al., 2020). Therefore, the effect of CD146 on DNA damage in brain metastases from lung cancer was examined in presence of pemetrexed. The results of immunofluorescence indicated γH2AX (a biomarker of DNA double strand breaks) foci were significantly more abundant in BM cells with CD146 knockdown in the presence of pemetrexed compared with control cells; meanwhile, the accumulation of γH2AX foci was reduced in PC9 cells with CD146 overexpression (Figure 3A). Similarly, immunoblot showed that CD146 downregulation enhanced DNA damage induced by pemetrexed in BM cells, and CD146 upregulation in PC9 cells with low CD146 expression attenuated this effect (Figure 3B). It has been shown that pemetrexed induces cell cycle arrest in NSCLC (Chen et al., 2014). According to FCM plots, we found that in the absence of pemetrexed, the expression level of CD146 did not overly affect cell cycle progression in brain metastases; however, after pemetrexed administration, the proportion of brain metastases with CD146 knockdown in the S phase was significantly increased compared with controls, while PC9 cells overexpressing CD146 attenuated the effect of pemetrexed (Figure 3C). This suggested that CD146 is required in enhanced DNA damage repair induced after treatment with pemetrexed. More interestingly, silencing or inhibition of CD146 in brain metastatic cells in the presence of pemetrexed downregulated cyclin A2 and CDK2 (cell cycle related proteins), whereas in PC9 cells, CD146 overexpression promoted their expression (Figure 3D). The latter findings suggested CD146 is critical for maintaining cell cycle progression after pemetrexed treatment.

### CD146 enhances PEM resistance in NSCLC brain metastatic cell lines via inhibition of apoptosis

**FIGURE 4:**
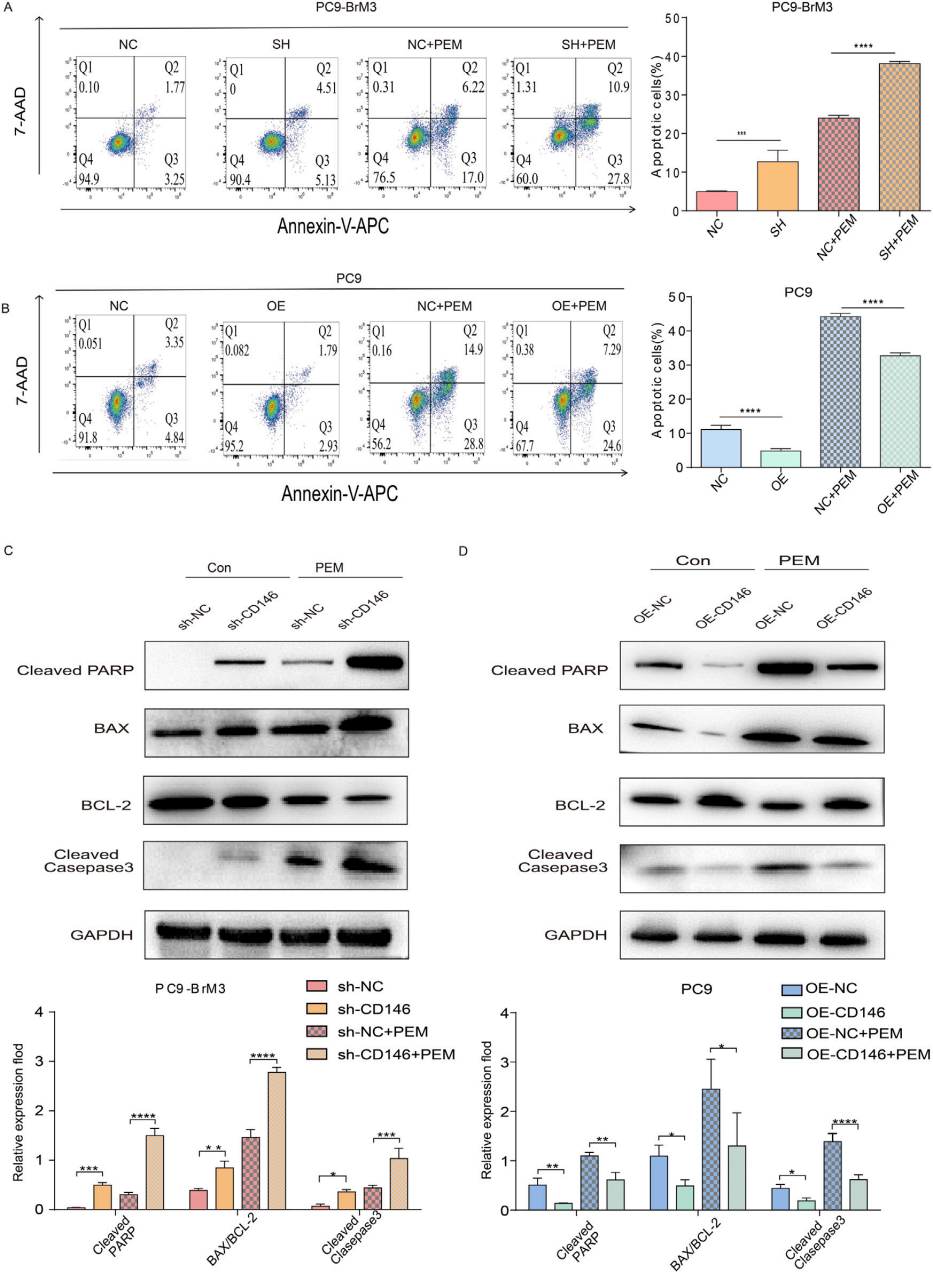
CD146 increases PEM resistance in PC9BrM3 cells by inhibiting apoptosis. **(A, B)** Evaluation of cell apoptosis by flow cytometry in CD146 silenced PC9BrM3 cells or CD146overexpressing PC9 cells treated with or without PEM (80 nM) for 72 h. **(C, D)** Immunoblot showing cleaved PARP, BAX, BCL2, cleaved caspase 3 protein amounts in CD146 knockdown PC9BrM3 cells or CD146overexpressing PC9 cells treated with or without PEM (80 nM) for 72 h (*P < 0.05, **P < 0.01, ***P < 0.001).

We previously demonstrated CD146 plays a critical regulatory role in pemetrexed dependent DNA and cell cycle arrest in lung cancer brain metastases. Therefore, we hypothesized that CD146 also mediates pemetrexed resistance in brain metastases through reduced apoptosis. Therefore, the effect of CD146 on apoptosis was assessed in lung cancer brain metastases by flow cytometry. It was clear that the apoptotic rate of brain metastases treated with pemetrexed after knocking down CD146 was significantly higher compared to control cells, with increased apoptotic cell ratio (Figure 4A); conversely, the apoptotic rate of PC9 cells overexpressing CD146 under pemetrexed treatment was higher than that of control cells (Figure 4B).

Moreover, the subsequent changes in protein amounts confirmed this finding. After treatment with pemetrexed, the expression levels of cleaved PARP, cleaved caspase 3 and BAX were markedly elevated in brain metastatic cells with CD146 knockdown compared with the control group while BCL2 was downregulated (Figure 4C); Conversely, after exposure to pemetrexed, the levels of cleaved PARP, cleaved caspase 3, and BAX were significantly reduced in PC9 cells overexpressing CD146 compared to the control group, whereas BCL2 exhibited an increase (Figure 4D). Echoing flow cytometry results, binding of pemetrexed to CD146 shRNA yielded the highest levels of cleaved PARP, cleaved caspase 3 and BAX, indicating that CD146 is resistant to pemetrexed mediated apoptosis in lung cancer brain metastases.

### CD146 enhances PEM resistance in NSCLC brain metastatic cell lines by inhibiting apoptosis via the NFκB pathway

**FIGURE 5:**
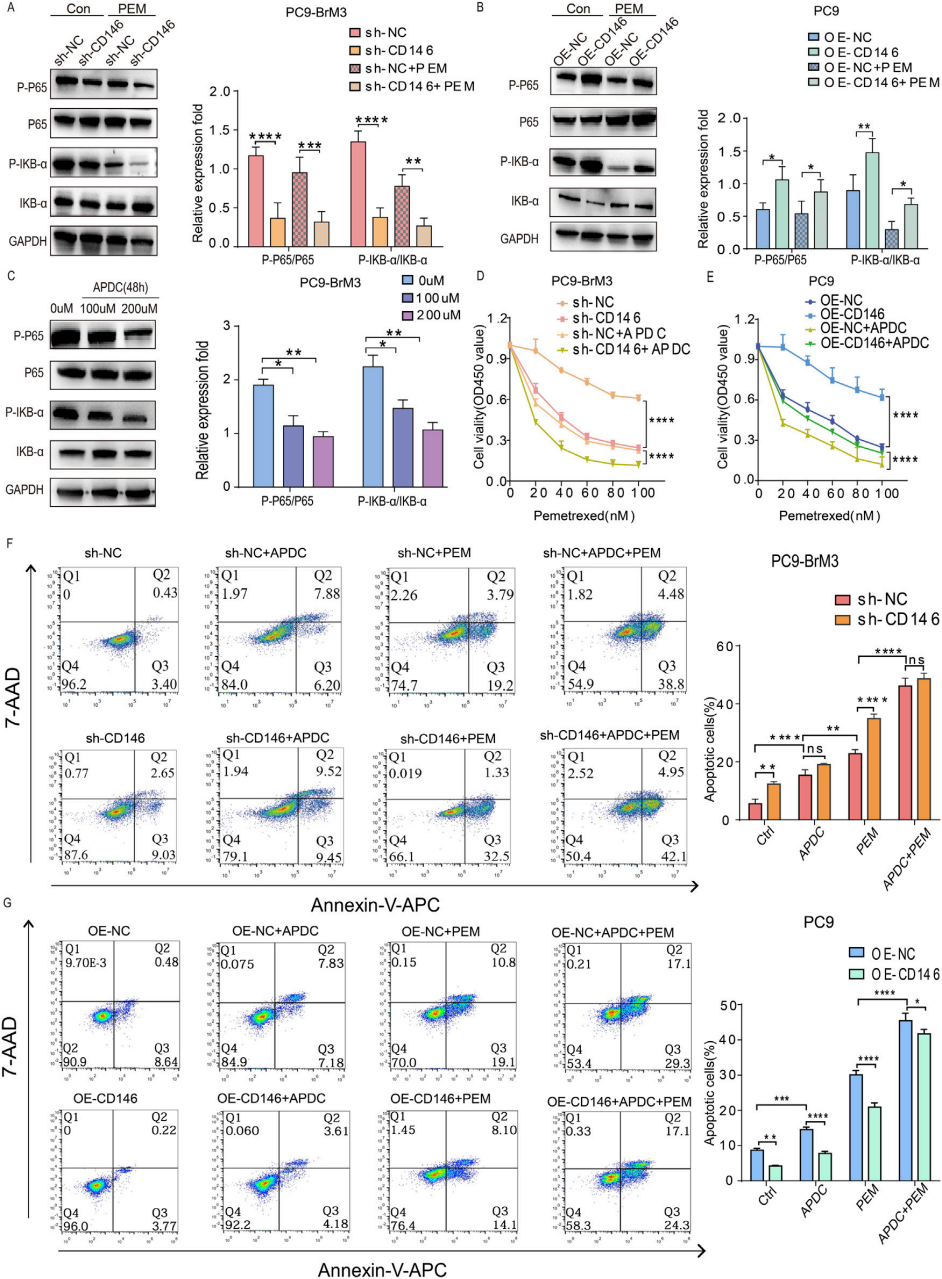
CD146 enhances PEM resistance in NSCLC brain metastatic cell lines by inhibiting apoptosis via NF-κB signaling. **(A, B)** Immunoblot showing pp65, p65, pIKBα and IKBα in CD146 knockdown PC9BrM3 cells or CD146overexpressing PC9 cells treated with or without PEM (80 nM) for 72 h. **(C)** PC9BrM3 cells were pretreated with various APDC doses for 48 h **(D, E)** CD146 knockdown PC9BrM3 or CD146 overexpressing PC9 cells were administered increasing PEM amounts in the presence or not of APDC (200 μM) for 72 h, followed by the CCK8 assay. **(F, G)** Flow cytometry analysis was employed to investigate cellular apoptosis in two distinct cell models: PC9BrM3 cells with CD146 knockdown and PC9 cells with CD146 overexpression. These cells were subjected to treatment with PEM (80 nM) alone or in combination with APDC (200 μM) (*P < 0.05, **P < 0.01, ***P < 0.001).

CD146 is known to induce the progression of malignant melanoma via the NFκB pathway (Ruma et al., 2016). We investigated whether CD146 induces pemetrexed resistance through this signaling pathway. First, immunoblot demonstrated pp65 and pIKBα were downregulated in brain metastases with knockdown CD146 relative to the control group, while pp65 and pIKBα were upregulated in PC9 cells overexpressing CD146 relative to the control group (Figures 5A, B). This indicates that CD146 could regulate NFκB signaling in lung cancer brain metastatic cells. More interestingly, the NFκB pathway was downregulated overall when pemetrexed exerted its effects, and pp65 and pIKBα were downregulated more significantly in brain metastatic cells with CD146 knockdown relative to controls. Similarly, pp65 and pIKBα levels in PC9 cells overexpressing CD146 did not decrease as much as in controls in the presence of pemetrexed (Figures 5A, B). Then, we used the NFκB inhibitor APDC to determine the appropriate concentration needed to artificially inhibit NFκB expression in PC9BrM3 cells (Figure 5C). Through the CCK8 assay, we found that inhibit of the NFκB pathway significantly increased the sensitivity of brain metastatic cells to pemetrexed, and interestingly, CD146’s effect on pemetrexed resistance was greatly reduced (Figures 5D, E). Flow cytometry analysis revealed that, compared to the individual addition of pemetrexed, the combination with the inhibitor APDC led to an increased apoptosis rate in BrM3 cells with knocked down CD146, as well as in PC9 cells with CD146 overexpression. This suggests that blocking the NFκB signaling pathway significantly enhances the sensitivity of brain metastatic cells to pemetrexed, while overexpression of CD146 attenuates this effect (Figures 5F, G). Based on these results, we speculate that CD146 could mediate pemetrexed resistance in brain metastatic cells by regulating NF-ΚB dependent resistance to apoptosis.

### Knockdown of CD146 enhances the anticancer effect of pemetrexed *in vivo*

**FIGURE 6:**
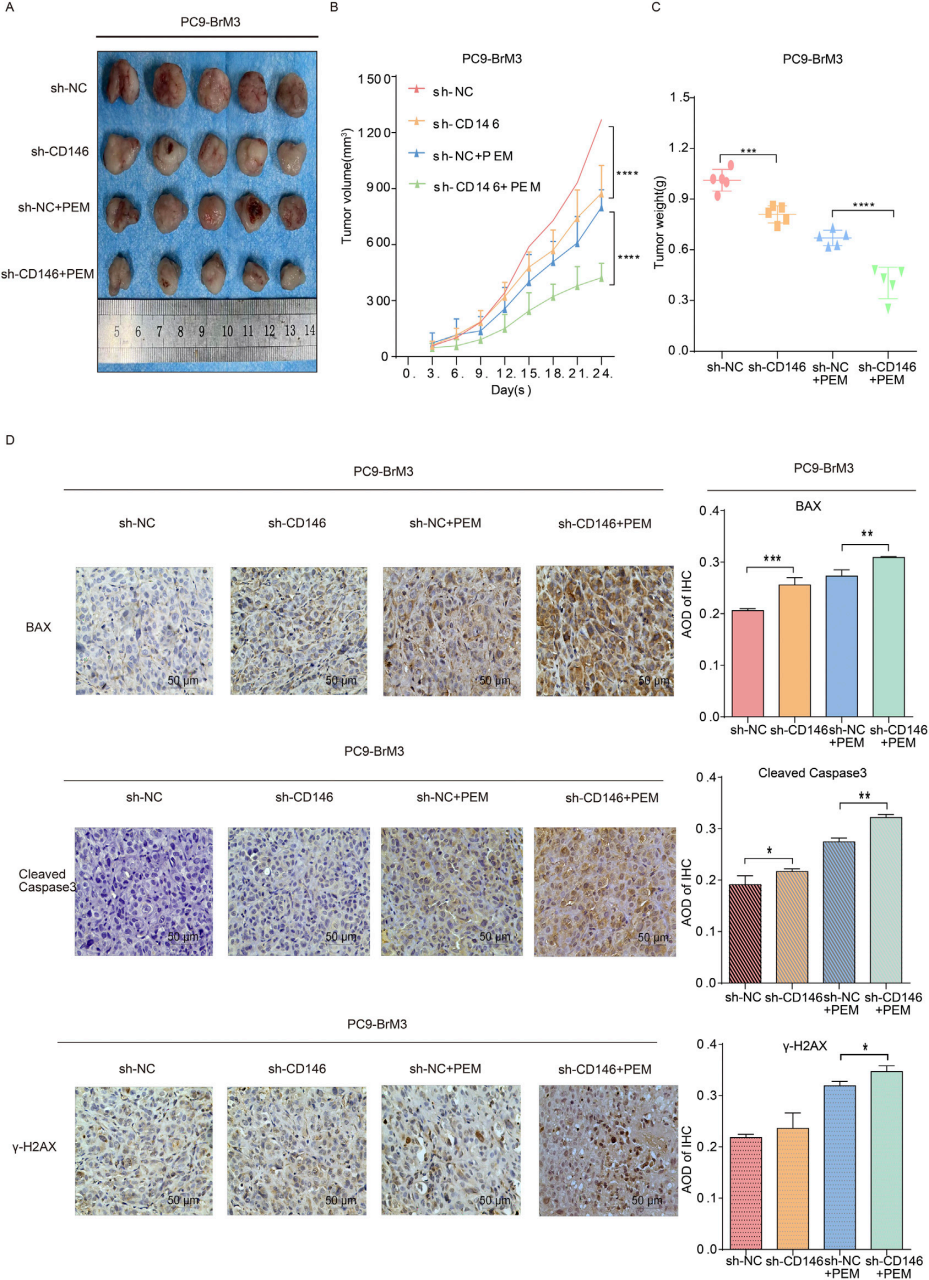
CD146 knockdown reverses chemoresistance of NSCLC brain metastases tumors *in vivo*. BALB/c nude mice underwent subcutaneous injection with PC9BrM3 cells with negative control shRNA transfection (BrM3shNC) or CD146 knockdown (shCD146) (5 × 10^6^ cells/mouse). Once the subcutaneous tumor reached 100,150 mm^3^, nude mice were intraperitoneally administered DMSO as control (Ctrl; n = 5, once weekly) or 100 mg/kg pemetrexed (PEM; n = 5, once weekly). **(A–C)** Representative photographs of tumors, tumor growth curves and tumor weights in various groups. **(D)** Representative micrographs for immunohistochemical assessment of BAX, cleaved caspase 3 and γH2AX proteins in CD146 silenced PC9BrM3 cells in the indicated groups (*P < 0.05, **P < 0.01, ***P < 0.001).

To further validate our *in vitro* findings, we established BrM3NC cell derived xenograft tumors and BrM3shCD146 cell derived xenografts in nude mice and applied them in combination with pemetrexed to assess pemetrexed resistance *in vivo*. We found their combined effect more significantly inhibited the growth of subcutaneous tumors in comparison with pemetrexed alone or knockdown CD146 alone; additionally, subcutaneous tumors were extracted from nude mice and weighed at the end of the study. As a result, tumors in the BrM3shCD146 cell group treated with pemetrexed had significantly lower weights compared with other experimental and control groups (Figures 6A–C). Immunoassays on subcutaneous tumors showed apoptosis related signature molecules, including BAX and cleaved caspase 3, and the DNA damage signature molecule γH2AX showed elevated levels in the BrM3shCD146 group in comparison with the BrM3NC group treated with pemetrexed (Figure 6D). These results suggested that CD146 may mediate pemetrexed resistance in NSCLC brain metastatic cell.

## Discussion

**FIGURE 7:**
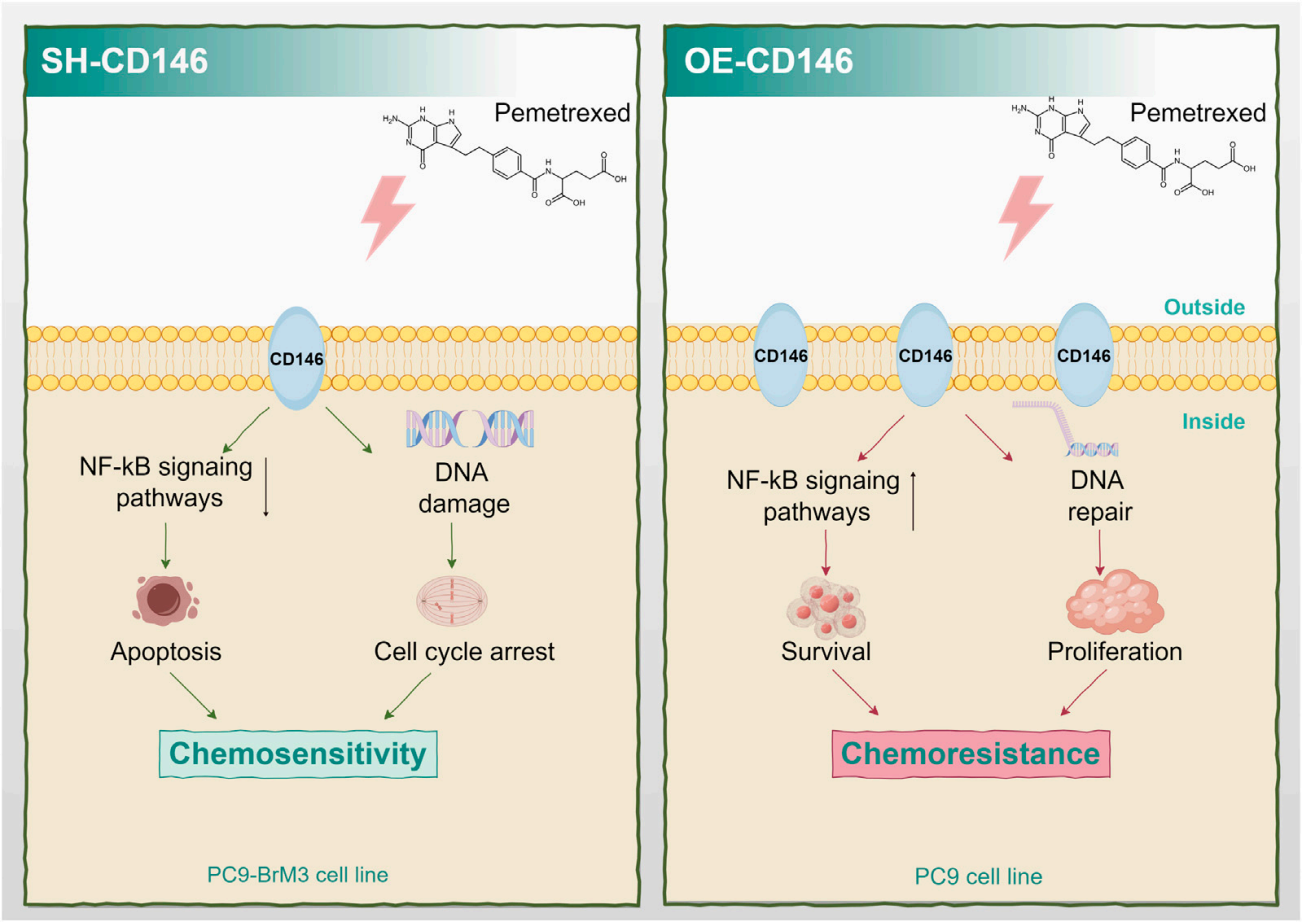
Schematic diagram of the mechanism by which high expression of CD146 confers PEM resistance in non-small cell lung cancer brain metastasis.

Current effective treatments for brain metastases from lung cancer combine surgical procedures, radiation therapy, chemotherapeutic approaches, molecular targeted therapy, and/or immunotherapy; however, treatment outcomes are particularly poor (Shi et al., 2017). Because indications for surgical and radiation therapies are limited (Yousefi et al., 2017; Suh et al., 2020), chemotherapy remains an indispensable therapeutic option for brain metastases. As a firstline chemotherapeutic agent for brain metastases from NSCLC, PEM remains the standard choice for patients. However, drug resistance is a crucial problem that cannot be ignored in the chemotherapy process, and the response of cases with BM to the drug is extremely low (Bailon et al., 2012; Barlesi et al., 2011; Arvanitis et al., 2020; Shah et al., 2020). It was demonstrated that lung cancer brain metastases are more resistant to pemetrexed compared with *in situ* lung cancer cells (Xu et al., 2020), Consistently, CD146 showed high expression in PC9BrM3 cells with PEM resistance as shown above. CD146 upregulated the NFκB signaling pathway and resistance to apoptosis, inducing resistance to PEM. Besides, CD146 promoted cell cycle progression and DNA repair, resulting in resistance to PEM (Figure 7).

CD146 plays an important role in many malignancies, e.g., breast and lung cancers, both in tumor progression and metastasis, which is closely associated with chemotherapy resistance (Tripathi et al., 2017; Liang et al., 2017; Zeng et al., 2020); its high expression is positively correlated with tumor drug resistance in multiple tumors. Additionally, CD146 contributes to the formation of the tumor microenvironment (TME) (Jing et al., 2023), influencing immune evasion and promoting angiogenesis and epithelial-mesenchymal transition (EMT), both of which are critical for tumor invasiveness and metastasis (Duan et al., 2021; Jiang et al., 2016; Ma et al., 2018). This highlights that CD146-mediated resistance to chemotherapy may be due to not only direct effects on tumor cells but also its regulatory role in the TME.

However, the correlation of CD146 with drug resistance in lung cancer brain metastasis remains undefined. As shown above, high CD146 expression can influence chemoresistance in brain metastases from lung cancer. Given the crucial role of CD146 in tumor progression and drug resistance, targeting CD146 represents a promising therapeutic strategy. Anti-CD146 antibodies or inhibitors may enhance chemosensitivity by attenuating NF-κB pathway activation and disrupting cell cycle progression. Early studies have shown that CD146-targeted therapies could potentially inhibit tumor invasion and metastasis (Ma et al., 2018). To overcome chemotherapy resistance, combination therapies involving CD146-targeted treatments are actively being explored. Combining chemotherapy with immune checkpoint inhibitors or molecular-targeted agents that block CD146 may enhance therapeutic efficacy. Recent studies suggest that such combinations may have synergistic effects in reducing tumor growth and resistance.

Chemotherapy resistance may occur via intrinsic or acquired mechanisms, including decreased intracellular drug accumulation, modified drug targets, and enhanced DNA repair (Zheng et al., 2019). In contrast, the efficacy of pemetrexed is mediated by the inhibition of DNA and RNA synthesis.

We found that downregulation of CD146 alone did not produce DNA damage and affect cell cycle progression, but under pemetrexed, downregulation of CD146 caused significant DNA damage and cell cycle arrest in PC9BrM3 cells, and overexpression of CD146 increased DNA repair and protected cell cycle progression in PC9 cells. This suggests that CD146 may influence the effect of pemetrexed on lung cancer brain metastatic cells through this general mechanism.

In addition, the effect of CD146 on apoptosis was examined. The above findings revealed PEM significantly increased the apoptotic rate of cancer cells. However, overexpression of CD146 attenuated this increase, while CD146 downregulation enhanced this increase.

CD146 is extensively involved in multiple oncogenic signal transduction pathways, including NFκB (Xue et al., 2022), VEGF/VEGFR (Jiang et al., 2012; Jouve et al., 2015), and PI3K/AKT (Xue et al., 2022). Nevertheless, studies on CD146 regulation of drug resistancerelated signaling pathways are not common. It was shown NFκB pathway induction not only causes resistance to apoptosis (Zheng et al., 2017), but is also associated with chemoresistance in a variety of cancers (He et al., 2021; Zhai et al., 2019; Zhao et al., 2018), although this has not been reported in lung cancer brain metastatic cells. The present data indicate CD146 regulates NFκB signaling and addition of a pathway inhibitor enhanced apoptosis and attenuated pemetrexed resistance in PC9BrM3 cells.

Although current studies suggest CD146 could mediate PEM resistance through increased DNA repair and resistance to apoptosis, the exact mechanisms by which CD146 mediates these effects are unclear, which deserves further attention. In addition, whether CD146 acts through other pathways or targets an enzyme or protein besides regulating the NFκB pathway to mediate PEM resistance is unknown, suggesting further related research is needed.

In summary, this study revealed that the resistance of NSCLC brain metastatic cells to PEM was dependent on CD146, which could mediate such resistance by attenuating PEMrelated DNA damage and cell cycle arrest in PC9BrM3 cells. In addition, CD146 mediates PEM resistance in PC9BrM3 cells via NFκB pathway activation to resist apoptosis. Therefore, CD146 is important in PEM resistance in NSCLC brain metastases and may be a therapeutic target to overcome chemoresistance and high CD146 expression in cases with refractory NSCLC brain metastases.

## Data Availability

The original contributions presented in the study are included in the article/supplementary material, further inquiries can be directed to the corresponding author.

## Abbreviation

APDC, Ammonium pyrrolidinedithiocarbamate; BM, brain metastases; CD146, cluster of differentiation 146; DHFR, dihydrofolate reductase; EMT, epithelialmesenchymal transition; FBS, fetal bovine serum; NFκB, Nuclear factor κB; NSCLC, non small cell lung cancer, PEM, pemetrexed; PI3K, phosphatidylinositol 3 kinase; AKT, protein kinase B; TS, thymidine synthasev; VEGF, vascular endothelial growth factor.
